# Tethered Cord Syndrome Associated With Lumbar Lipomyelomeningocele: A Case Report

**DOI:** 10.7759/cureus.22590

**Published:** 2022-02-25

**Authors:** Ahmed Harazeen, Neeharika Thottempudi, Joseph Sonstein, Xiangping Li, Laura Wu, Prashant Rai, Todd Masel

**Affiliations:** 1 Neurology, University of Texas Medical Branch, Galveston, USA; 2 Urology, University of Texas Medical Branch, Galveston, USA

**Keywords:** urinary incontinence, spinal dysraphism, spinal bifida, tethered cord syndrome, lipomyelomeningocele

## Abstract

The objective is to describe a rare case of lumbar lipomyelomeningocele presenting as progressive urinary incontinence. Lipomyelomeningocele is a type of closed spinal dysraphism typically presenting as a lipomatous mass contiguous with a neural defect above the gluteal crease. Tethered cord syndrome is defined as symptoms and signs caused by excessive spinal cord tension from an abnormally low conus medullaris, with an abnormally thick filum terminale attached to the lower sacral region. A 19-year-old male with no remarkable medical history presented with low back pain and urinary incontinence for the past one year. On physical exam patient had normal motor strength, sensory testing to all modalities was intact. The rectal tone was normal, and no saddle anesthesia was noted. MRI lumbar spine revealed lumbar lipomyelomeningocele with associated tethered cord syndrome. The patient underwent tethered cord release surgery with lipoma excision. Pathology of the soft tissue showed fibrovascular tissue and mature adipose tissue consistent with lipoma. The majority of cases of tethered cord syndrome are related to spinal dysraphism, a rare pediatric syndrome. It is potentially treatable if caught early, and MRI can help with an accurate diagnosis of the condition. Older adults are more likely to present with urological and neurological complaints. Surgical un-tethering is indicated in patients with progressive symptoms. In our case, the only presenting symptom was urinary incontinence, and the neurological exam was normal other than lower lumbar paraspinal tenderness.

## Introduction

Lipomyelomeningocele is a rare congenital type of closed spinal dysraphism that typically presents as a lipomatous mass contiguous with a neural defect. It is usually located in the lumbar region above the gluteal crease and is found in 0.3 and 0.6 per 10,000 live births [1-3]. A commonly reported finding is a lipomatous mass that is clinically present at birth located at the lumbosacral region positioned at the midline. Other cutaneous manifestations, such as hair, nevus, hemangioma, and skin dimples, can also be present [1,2]. Unlike typical cases of lipomyelomeningocele which are symptomatic after birth, our patient started having symptoms during adulthood. Initial clinical diagnosis of lipomyelomeningocele can be challenging, as 48% of affected patients have been found to have a normal neurological exam. There should always be a high index of suspicion for tethered cord syndrome to order radiological assessment as it will guide diagnosis and treatment [4]. Other imaging modalities may include ultrasonography, although its usefulness is dependent upon operator experience and the position of the fetus [5-7]. The gold standard for diagnosis is MRI of the spine [6,7].

## Case presentation

**Figure 1:**
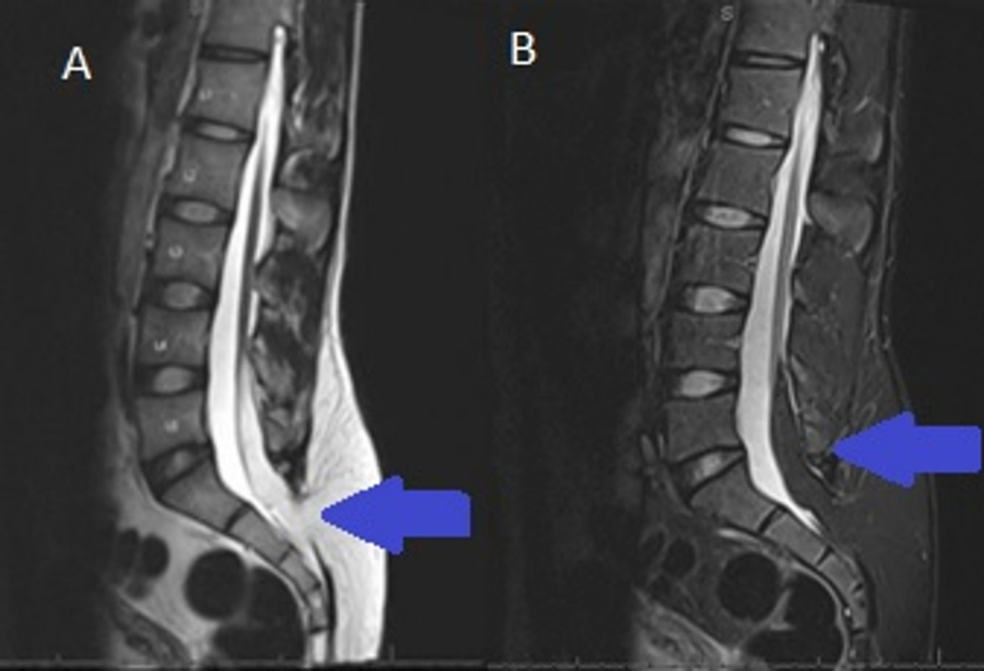
Lumbar spine sagittal view showing findings of tethered spinal cord with associated posterior sacral defect and lipomyelomeningocele MRI lumbar spine (A) T1 sequence and (B) T2 sequence show posterior sacral defect and lipomyelomeningocele (blue arrow).

A 19-year-old right-handed Hispanic Spanish-speaking male with no prior past medical history presented to the hospital with lower back pain, urinary incontinence for one year. He has been wearing diapers daily. He denied any motor weakness or paresthesias in extremities, bowel incontinence, or saddle anesthesia. On physical exam his mental status assessment and cranial nerve examination were normal. The sensation was intact to all modalities throughout, and motor strength was normal. Deep tendon reflexes were 2+ throughout and no pathological reflexes were noted. The rectal tone was intact and there was no saddle anesthesia. He did have a circular bump in his lumbar region measuring approximately 10 cm x 10 cm, with no skin dimple or tuft of hair. In the emergency department, over 1,200 mL of residual urine was removed through a straight catheter. MRI of the lumbar spine revealed a low-lying conus, thickened filum, and a fibrolipoma inserting into a terminal lipoma continuous with subcutaneous fat through a defect in the sacrum (Figures 1A, 1B). He was diagnosed with lumbar lipomyelomeningocele with associated tethered cord syndrome. CT abdomen/pelvis showed trabeculation and marked circumferential wall thickening of the urinary bladder trabeculation associated with left greater than right hydronephrosis and left renal atrophy consistent with neurogenic bladder. CT lumbar spine showed incomplete fusion of the posterior elements of L5, terminal lipoma contiguous with subcutaneous fat through dorsal dysraphism in the posterior sacrum. Neurosurgery was consulted and they performed lumbar osteoplastic laminoplasty of L2-L4, resection of intramedullary lipoma, resection of filum terminale, the release of tethered cord, and resection of 6 cm diameter subcutaneous lipoma. The filum terminale was dissected from the underlying nerve roots, and intraoperative stimulation did not produce any significant responses to the perianal sphincters and the lower limb muscles. The patient did undergo intraoperative somatosensory and motor evoked potential monitoring, which did not reveal any reduction in the evoked potentials intermittently. Pathology of the soft tissue showed fibrovascular tissue and mature adipose tissue consistent with lipoma. Postoperative MRI did show post-surgical changes of L4-S1 laminotomy for resection of lipoma with associated hematoma seen at the surgical bed. Despite surgery, the patient’s symptoms did not improve. Urology was also consulted and recommended self-catheterization at home using clean intermittent catheterization every six hours. The patient did follow up with urology in the clinic; however, unfortunately, patient did not complete urodynamics or other bladder diagnostic evaluations.

## Discussion

Lipomyelomeningocele is a form of closed neural tube defect and it can present with neurological deficits secondary to a tethered cord [5]. The urinary dysfunction likely results from impaired innervation of the urinary system, either from malformation during embryogenesis or from a tethered cord caused by the lipomyelomeningocele [5]. Urinary complaints can be also due to detrusor paresis, external sphincter dysfunction, or detrusor sphincter dyssynergy. The condition is potentially treatable if caught early, and MRI can help with an accurate diagnosis. Surgical un-tethering is indicated in patients with progressive symptoms. Surgical correction when the patient reaches one month of age or at the time of diagnosis provides a degree of reversibility not seen in older children [3,5]. Although the initial signs and symptoms commonly present at birth, our patient presented in adulthood with urinary incontinence for one year. In this case, there was no weakness, no numbness, and no autonomic or sexual dysfunction. MRI of the lumbar spine revealed a lumbar lipomyelomeningocele with tethered cord syndrome. In our case, the only presenting symptom was bladder incontinence, and the neurological exam was normal other than lower lumbar paraspinal tenderness.

Our patient did undergo surgical un-tethering, although the surgery, unfortunately, did not improve the patient’s symptoms. Patients who primarily have motor or sensory deficits are more likely to improve following surgery compared to those with urinary or sexual dysfunction. Urological dysfunction appears to be more reversible in younger patients, especially infants [4]. Complications following surgery include CSF leakage, recurrent urinary tract infection, hydronephrosis, nerve injury, incomplete wound healing, infection, and meningitis. Surgical outcomes for lipomyelomeningocele are dependent on the age of the patient at the time of diagnosis as well as preoperative function [1]. Studies show that about 92% of children have no neurological symptoms, have normal exams, and undergo surgery uneventfully with no complications [4,5]. None of the children with preoperative bowel or bladder symptoms, however, had complete recovery despite improvement in sensory and motor deficits [1].

## Conclusions

In this report, we discussed a rare case of lipomyelomeningocele presenting in adulthood with a one-year history of urinary incontinence. No other neurological symptoms were present, and the neurological exam was normal aside from lower lumbar paraspinal tenderness. MRI proved essential in making the diagnosis. This case highlights the importance of MRI in detecting the condition, and it should be noted that early diagnosis is important for improving outcomes of surgical treatment. Early surgery is indicated to prevent cord infarction, paraplegia, and bowel and bladder dysfunction.
